# Estimating dengue burden among family contacts through cluster investigation around probable cases in 2022 and 2023 in the Central Region, Burkina Faso

**DOI:** 10.1186/s40249-024-01212-5

**Published:** 2024-06-12

**Authors:** Jean Claude Romaric Pingdwindé Ouédraogo, Sylvain Ilboudo, Prosper Bado, Tegwindé Rebeca Compaoré, Alix Tougma, Mathieu Nitiéma, Abdou Azaque Zouré, Lazare Belemnaba, Sylvin Ouédraogo, Léon Gueswendé Blaise Savadogo

**Affiliations:** 1 Drug Development Laboratory, African Centre of Excellence for Training, Research and Expertise in Drug Sciences, (LADME/CEA-CFOREM), Joseph Ki-Zerbo University, Ouagadougou, Burkina Faso; 2 Phytomedicines and Medicines Research and Development Laboratory (LR-D/PM), Research Institute for Health Sciences (IRSS)/ CNRST, Ouagadougou, Burkina Faso; 3 International Research Laboratory - Environment, Health, and Societies (IRL 3189, ESS), CNRST, Ouagadougou, Burkina Faso; 4 Pietro Annigoni Biomolecular Research Centre (CERBA), Ouagadougou, Burkina Faso; 5 Research Laboratory for Infectious and Parasitic Diseases (LR/MIP), Research Institute for Health Sciences (IRSS)/ CNRST, Ouagadougou, Burkina Faso; 6 Joseph KI-ZERBO University/University Centre of Ziniaré, Ziniaré, Burkina Faso; 7 Higher Institute for Health Sciences, Nazi Boni University, Bobo-Dioulasso, Burkina Faso

**Keywords:** Dengue, Index case, Contact, Asymptomatic, Subclinical, Symptomatic, Burkina Faso

## Abstract

**Background:**

In 2023, Burkina Faso experienced the largest dengue epidemic ever in Africa. This study aimed to estimate the prevalence of symptomatic, subclinical, and asymptomatic dengue and determine the associated factors among adult contacts of dengue in the Central Region, Burkina Faso.

**Methods:**

This cross-sectional study included contacts of dengue probable cases through cluster sampling in 2022–2023. These suspected cases that tested positive were identified from the five health facilities (Pissy CMA, Saaba CM, Kossodo CMA, Samandin CM, and Marcoussis CSPS) that reported the highest number of cases in 2021 per district. All participants underwent dengue and malaria rapid diagnostic tests (RDT). Samples positive for non-structural 1 protein antigen (AgNS1) and/or immunoglobulin M (IgM) were tested for serotype detection by reverse transcription polymerase chain reaction (RT-PCR). Binary logistic regression was done to identify the determinants of asymptomatic, subclinical, and symptomatic dengue among contacts of probable dengue cases.

**Results:**

A total of 484 contacts were included, mostly in 2023 (75.2%). Most participants were females (58.6%), residing (24.3%) and passing their daytime (23.1%) in Saaba. The overall prevalence of dengue was estimated at 15.1% [95% confidence interval (*CI*): 12.0–18.6%], representing cases not seeking care in hospitals. Asymptomatic cases represented 2.9% (95% *CI*: 1.6–4.8%). Subclinical and symptomatic cases accounted for 6.0% (95% *CI*: 4.1–8.5%) and 6.2% (95% *CI*: 4.2–8.7%), respectively. Of the 58 samples tested by RT-PCR, 10 were confirmed for serotype 3 in 2023. Malaria cases were estimated at 5.6% (95% *CI*: 3.7–8.0%). After adjustment, participants claiming that a virus transmits dengue were likelier to have asymptomatic dengue [adjusted odds ratio *(*a*OR)* = 7.1, 95% *CI*: 2.4–21.0]. From the multivariable analysis, subclinical dengue was statistically associated with being included in the study in 2023 (a*OR* = 30.2, 95% *CI*: 2.0–455.5) and spending the daytime at Arrondissement 4 (a*OR* = 11.5, 95% *CI*: 1.0–131.0). After adjustment, symptomatic dengue was associated with living less than 50 m away from cultivated land (a*OR* = 2.8, 95% *CI*: 1.1–6.9) and living less than 50 m from a stretch of water (a*OR* = 0.1, 95% *CI*: 0.0–0.6).

**Conclusions:**

The overall burden of dengue among populations not seeking care in hospitals was quite high, with few asymptomatic cases. Efforts to manage dengue cases should also target non-hospital cases and raise population awareness. The 2023 epidemic could be due to dengue virus (DENV)-3.

**Graphical Abstract:**

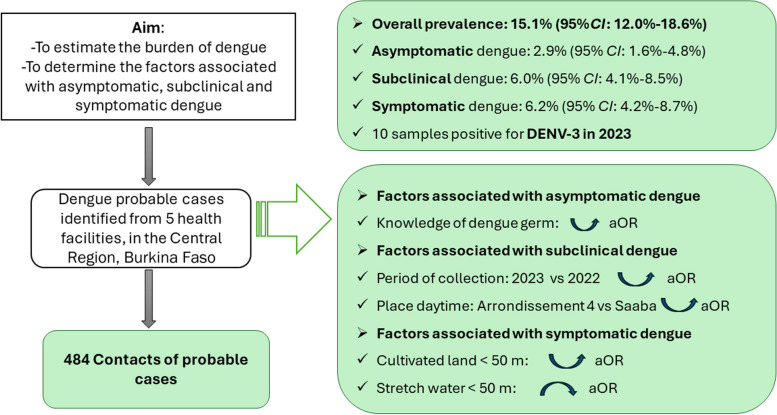

## Background

Dengue is one of the most widespread arthropod-borne viral diseases, menacing about half of the world's population [1]. Global estimates show 390 million cases annually, with good evidence of dengue in at least 128 countries [2]. The World Health Organization (WHO) regions of America, Asia, and Africa are the most affected by dengue [1]. Estimates give a similar burden in Africa than in America, with 16 (11–22) million versus 13 (9–18) million [3]. However, due to poor surveillance, dengue cases have been underreported in Africa [4, 5].

At least 58% of African countries reported dengue already [2]. East Africa has been most affected, followed by West Africa [2, 6]. Since 2014, studies have highlighted that West Africa is becoming a new front for dengue fever [7]. In Burkina Faso, dengue fever was first reported in 1925, and cases were later reported in 1982 [8, 9]. The subsequent studies confirmed that it is endemic-epidemic in the country, with the circulation of all 4 serotypes [5, 10–12]. According to data from passive surveillance, health districts in the Central region are the most affected by dengue in the country [13]. In 2023, Burkina Faso experienced the largest epidemic in Africa, with 154,867 suspected cases, 70,433 probable cases and 709 deaths [14]. As of March 3, 2024, the country reported a total of 164,848 suspected cases, including 73,497 probable and confirmed cases, with a case fatality rate of 0.45% (over suspected cases) [15].

According to the WHO, dengue cases are more asymptomatic or mild than symptomatic [1]. In a systematic review, asymptomatic dengue prevalence was pooled at 59.3% (95% confidence interval (*CI*): 43.8–74.8%), higher during outbreaks than outside outbreak periods [16]. However, most studies included in that review were done in Asia and South America. On the contrary, a systematic review that included one study from Africa claimed a prevalence of 0.2% among healthy populations [17]. Using studies published in Africa between 2000 and 2019, another systematic review found a lower prevalence of all markers among asymptomatic cases than symptomatic dengue [18]. Thus, the pooled prevalence of asymptomatic dengue was 0.0% (95% *CI*: 0.0–0.5%), 3.5% (95% *CI*: 0.8–7.8%) and 15.6% (95% *CI*: 9.9–22.2%), respectively for ribonucleic acid (RNA), immunoglobulins (Ig)M and IgG [18]. Among febrile populations, dengue prevalence reached 24.8% (95% *CI*: 13.8–37.8%), 10.8% (95% *CI*: 3.8–20.6%) and 8.4% (95% *CI*: 3.7–14.4%), respectively, for IgG, IgM, and RNA [18].

Data on the actual extent of the different types of dengue in Africa is therefore scarce. In Burkina Faso, most studies included data of febrile participants from hospitals or laboratories [10, 19–21]. However, these data do not give the true burden of dengue as not all symptomatic cases would seek care at health facilities, nor the asymptomatic cases. To break the transmission and control of dengue in Burkina Faso, a big challenge is to estimate the actual burden of dengue in the area through household-based data.

This study aimed to estimate the prevalence of symptomatic, subclinical, and asymptomatic dengue fever and determine the associated factors among adult contacts of probable cases through a cluster sampling in the Central Region, Burkina Faso.

## Methods

### Study design

This study is part of an overall research aiming to estimate the prevalence of symptomatic and asymptomatic dengue among adults in Burkina Faso in 2022 and 2023 [22]. The overall study included two populations, dengue suspected cases from selected health facilities and the contacts of those turning probable cases. Within this research, we conducted cluster sampling through household-based data collection, systematically targeting contacts of dengue probable index cases. Data were collected from September to November 2022 and September to November 2023, at the end of the rainy season when malaria and dengue cases peak.

### Study setting

This study was conducted in Burkina Faso. It targeted the Central Region with the Capitale City of Ouagadougou surrounded by the six rural municipalities of Koubri, Saaba, Pabré, Komsilga, Komki-Ipala, and Tanghin-Dassouri [23]. Ouagadougou is structured into 12 arrondissements and 55 sectors, with 2,415,266 inhabitants in 2019 [24]. According to the same population and housing census, the Central Region had a population of 3,032,668 inhabitants, with 62.4% of adults [25].

The regional health system includes 5 health districts, which are Bogodogo, Baskuy, Boulmiougou, Sig-Nonghin and Nongr Massom health districts.

### Sample size estimation

We determined the minimum sample size using the formula for estimating a single proportion [26]:

$$\text{n }\ge \frac{{Z}_{1-\propto /2}^{2} X p(1-p)}{{e}^{2}}$$

-p: anticipated prevalence of asymptomatic dengue fever;

-Z_1-α/2_: percentage standard deviation corresponding to the two-sided significance level. For α = 5%, Z_1-α/2_ = 1.96.

-e: precision of 1.75% (half of 3.5%, the prevalence of immunoglobulin M in Africa).

We assumed that the contacts would be surveyed late, so the prevalence of immunoglobulin M was used to calculate the sample size. It was pooled at 3.5% (95% *CI*: 0.8–7.8%) among apparently healthy people in Africa [18]. The required sample size was estimated at 424, then adjusted to 472, considering an adjustment of 10% non-response rate with the formula (n = $\frac{{n}_{0}}{1-{n}_{r}}$).

### Participants characteristics

The study considered individuals aged 16 years and older, family contacts of dengue probable cases, regardless of the presence of fever or other symptoms. Mentally debilitated individuals were excluded from the study. Participants were further classified as follows based on clinical and serological characteristics:

**Dengue fever suspected case**: fever (≥ 38.5 °C) in the last five days with at least 2 of the following symptoms (headache, retro-orbital pain, myalgias, arthralgias, skin rash, bleeding manifestations, or shock syndrome) [27].

**Probable dengue fever:** a suspected case with a positive rapid diagnostic test (RDT) for dengue non-structural 1 protein antigen (AgNS1) and/or immunoglobulins (Ig)M and/or IgG**.** Probable cases identified from the health facilities were the index cases. Probable cases identified among the contacts of index cases were referred to as symptomatic cases. These symptomatic cases are undiagnosed infection [17] that did not seek care for the ongoing episode in a health facility. Without antibody titers, we did not consider distinguishing primary and secondary infections.

**Subclinical dengue case**: presenting a fever and/or other symptoms but not fitting the definition of a suspected case. It is sometimes referred to as mild infection, as considered in some studies.

**Asymptomatic dengue case:** participants turning positive for dengue RDTs with positive AgNS1 and/or IgM markers without any fever or symptom, fitting clinically undetectable infections [17].

**Dengue contacts of an index case**: participant sharing the same household, house, or compound (Celibateriums in French) with an index case.

### Data collection

We conducted face-to-face interviews with a structured questionnaire and RDTs, using the Kobo Toolbox. The questionnaire included participants-level factors (sociodemographic characteristics and knowledge of dengue) and household-level factors (environmental characteristics).

Index cases were identified through a three-stage sampling [22]. These symptomatic participants who were at least positive for AgNS1 and/or IgM were followed home. Then, all people residing in the same household or compound as the dengue probable case were asked to participate in the study. Participants were tested for malaria and dengue using RDTs. The SD Bioline Malaria Ag P.f kit (SD Standard Diagnostics, INC., Republic of Korea) and the Standards Q Malaria P.f Ag kit (SD Biosensor, Inc., Republic of Korea) were used to test for malaria by *Plasmodium falciparum*. The WONDFO Dengue NS1/IgG/IgM kit (Guangzhou Wondfo Biotech Co., Ltd, China) was used to test for the infection with the dengue virus. The tests followed the manufacturers procedures. Participants at least positive for AgNS1 and/or IgM were sampled for serotyping.

Participants positive for dengue RDT without presenting any symptom or sign were contacted up to the following 10 days to confirm they were not presymptomatic.

### Characterization of dengue viruses serotypes

#### Extraction of dengue viral RNA

Dengue virus RNA extraction was performed using the QIAamp Viral RNA Mini Kit (Qiagen, Germany), according to the manufacturer protocol. The extract was stored at -80 °C for molecular testing.

#### Serotyping of dengue viruses

Identification of the dengue virus genotypes was performed using Sacace PCR kit for detection (Sacace Biotechnologies, Italy). Differentiation of Dengue virus genotypes 1, 2, 3 and 4 in clinical material Dengue Real-™ Genotype (Sacace, Como, Italy) and the PCR steps followed the manufacturer instructions. This resulted in a total reaction volume of 25 µl for each PCR. To guarantee the quality of the results, three controls, which are negative control of extraction (NCE), positive control of amplification (C +) and negative control of amplification (NCA) were used.

The PCR reaction mixtures contained in sterile 0.2 ml microtubes were introduced onto the SaCycler-96 Real-Time PCR v.7.3 plate (Sacace Biotechnology, Italy) for amplification. The amplification program consisted of one cycle of 50 °C for 30 min, 95 ˚C for 15 min, followed by five cycles of 95 ˚C for 10 s, 56 ˚C for 40 s, and 72 ˚C for 20 s, and finally 40 cycles of 95 ˚C for 10 s, 54 ˚C for 40 s, and 72 ˚C for 20 s.

The results were interpreted using the RealTime_PCR software v7.9 (Dna -Technology LLC, Moscow, Russia) by the crossing or not crossing of the threshold line by the fluorescence curve. The genotypes were then identified following the manufacturer’s protocol.

### Statistical analyses

The software STATA/IC 16.1 (StataCorp LLC, College Station, Texas 77,845 USA) was used for analyses.

The prevalence of asymptomatic, subclinical, and symptomatic dengue fever was estimated with 95% confidence intervals. They were presented for the overall sample, symptomatic cases, subclinical cases, and asymptomatic cases. Independent variables were presented with mean ± standard deviation (SD) or median [interquartile range (IQR)] for the quantitative variables. Qualitative variables were presented with frequency and percentage.

Further, a binary logistic regression was done to identify the determinants of asymptomatic, subclinical, and symptomatic dengue among contacts of probable dengue cases. A univariate binary logistic regression was first done to identify the sociodemographic and environmental factors associated with asymptomatic, subclinical, and symptomatic dengue. Then, factors significant at 20% and those pertinent (like age and sex) even not significant in the univariate logistic regression were included in the multivariable analysis. The model with the lowest Akaike Information Criteria (AIC) and the narrowest intervals was retained for each dengue type.

## Results

### Overall dengue prevalence

Details of dengue prevalence among the 484 included participants are presented in Table 1.

**Table 1:** Dengue prevalence and markers among family contacts

Characteristics	*n* (%)	Prevalence, % (95% *CI*)
**Overall sample**		
**No dengue**	**411 (84.9)**	
IgG	30/411 (34.1)	
**Overall dengue**	**73 (15.1)**	15.1 (12.0–18.6)
IgG	36 (49.3)	
IgM	34 (46.6)	
IgG + IgM	21 (28.8)	
AgNS1 + IgM	7 (9.6)	
AgNS1	5 (6.8)	
***Plasmodium falciparum***	**27 (5.6)**	5.6 (3.7–8.0)
**Coinfection dengue-malaria**	**10 (2.1)**	
**Asymptomatic dengue**	**19 (2.9)**	2.9 (1.6–4.8)
IgM	7 (36.8)	
IgM + IgG	6 (31.6)	
AgNS1 + IgM	1 (5.3)	
Coinfection with malaria	0 case	
**Subclinical dengue**	**29 (4.1)**	6.0 (4.1–8.5)
IgM	18 (62.1)	
IgM + IgG	8 (27.6)	
AgNS1	3 (10.3)	
Coinfection with malaria	5 cases	
**Symptomatic dengue**	**30 (6.2)**	6.2 (4.2–8.7)
IgM	9 (30.0)	
IgG	6 (20.0)	
IgM + IgG	7 (23.3)	
AgNS1 + IgM	6 (20.0)	
AgNS1	2 (6.7)	
Coinfection with malaria	5 cases	

*AgNS1* Non-structural 1 protein antigen, *IgM* Immunoglobulins M, *IgG* Immunoglobulins G

The overall prevalence of dengue was estimated at 15.1% (95% *CI*: 12.0% –18.6%). Isolated or associated immunoglobulin M predominated among dengue cases. All cases with symptoms (subclinical and symptomatic) accounted for 12.6% (9.7% –15.9%). The ratio of asymptomatic to symptomatic was 0.5:1 (14/30). The ratio of asymptomatic to all cases with symptoms was 0.2:1.

The RT-PCR tests performed on 58 samples positive to AgNS1 and/or IgM in 2022–2023 confirmed 10 participants positive for serotype 3 in 2023. They were either symptomatic (4 positive for AgNS1 + IgM, 2 for IgM and 1 for AgNS1) or subclinical (2 positive for AgNS1 and 1 for IgM).

Among the dengue-negative participants, 34.1% were positive for IgG but could be past primary dengue or secondary early cases.

Overall, *Plasmodium falciparum* cases represented 5.6% (95% *CI*: 3.7% – 8.0%).

### Asymptomatic dengue prevalence

Considering all participants positive for dengue RDT, 19.2% (14/73) were asymptomatic. From the overall sample, asymptomatic cases represented 2.9% (95% *CI*: 1.6% –4.8%). There was no coinfection with malaria.

### Subclinical dengue prevalence

Of the 73 dengue cases, subclinical cases accounted for 39.7%. Subclinical dengue was estimated at 6.0% (95% *CI*: 4.1%–8.5%). Five (5) subclinical cases associated with malaria.

### Symptomatic dengue prevalence and markers

Among positive cases, 41.1% were symptomatic. In the overall sample size, symptomatic dengue was estimated at 6.2% (95% *CI*: 4.2%–8.7%). In addition, 5 cases were also positive for malaria.

### Sociodemographic features of the participants

The sociodemographic characteristics are presented below in Table 2. For the different types of dengue, most participants were included from the epidemic year 2023. Most asymptomatic cases came from Saaba Medical Centre (CM) (6/14), and most subclinical (72.4%) and symptomatic (50.0%) cases from Pissy Medical Centre with Surgical Antenna (CMA), respectively. Among the uninfected participants and subclinical cases, half were aged more than 28 years (median = 28 years). Half of the asymptomatic cases were older than 35, while half of symptomatic cases aged more than 23 years. Only asymptomatic cases were dominated by males (57.1%).

**Table 2:** Distribution of sociodemographic features of the participants according to the type of dengue

Variables		Non dengue cases (*n* = 411) *n* (%)	Asymptomatic cases (*n* = 14) *n* (%)	Subclinical cases (*n* = 29) *n* (%)	Symptomatic cases (*n* = 30) *n* (%)
**Period of collection**					
Non-epidemic year (2022)		102 (24.8)	1 (7.1)	1 (3.5)	0 (0.0)
Epidemic year (2023)		309 (75.2)	13 (92.9)	28 (96.6)	30 (100.0)
**Health facilities**					
Pissy CMA		90 (21.9)	3 (21.4)	21 (72.4)	15 (50.0)
Saaba CM		107 (26.0)	6 (42.9)	4 (13.8)	8 (26.7)
Kossodo CMA		119 (29.0)	4 (28.6)	3 (10.3)	4 (13.3)
Samandin CM		84 (20.4)	1 (7.1)	1 (3.5)	2 (6.7)
Marcoussis CSPS		11 (2.7)	0 (0.0)	0 (0.0)	1 (3.3)
**Age, Years**	^a^Min–Max	16–90	19–65	16–69	16–57
	^b^Mean ± SD	32.6 ± 14.3	37.9 ± 16.1	31.2 ± 13.4	28.9 ± 12.6
	^c^Median (IQR)	28 (22–42)	35 (23–57)	28 (23–34)	23 (20–39)
**Sex**					
Female		241 (58.6)	6 (42.9)	22 (75.9)	20 (66.7)
Male		170 (41.4)	8 (57.1)	7 (24.1)	10 (33.3)
**Education level**					
No education		83 (20.2)	1 (7.1)	9 (31.0)	6 (20.0)
Primary		67 (16.3)	3 (21.4)	3 (10.3)	3 (10.0)
Secondary		186 (45.3)	4 (28.6)	12 (41.4)	12 (40.0)
Tertiary		75 (18.3)	6 (42.9)	5 (17.2)	9 (30.0)
**Marital status**					
Never married		181 (44.0)	5 (35.7)	15 (51.7)	18 (60.0)
Currently married		200 (48.7)	7 (50.0)	11 (37.9)	9 (30.0)
Previously married		30 (7.3)	2 (14.3)	3 (10.3)	3 (10.0)
**Main occupation**					
Student		111 (27.0)	4 (28.6)	10 (34.5)	9 (30.0)
Housewife		78 (19.0)	2 (14.3)	7 (24.1)	7 (23.3)
Private employee		70 (17.0)	4 (28.6)	5 (17.2)	6 (20.0)
Trader		58 (14.1)	0 (0.0)	3 (10.3)	1 (3.3)
Public servant		33 (8.0)	2 (14.3)	0 (0.0)	2 (6.7)
Unemployed		26 (6.3)	1 (7.1)	4 (13.8)	2 (6.7)
Other occupations		35 (8.5)	1 (7.1)	0 (0.0)	3 (10.0)
**Residence**					
Saaba		100 (24.3)	5 (35.7)	4 (13.8)	7 (23.3)
Arrondissement 6		63 (15.3)	3 (21.4)	7 (24.1)	6 (20.0)
Arrondissement 10		68 (16.6)	3 (21.4)	2 (6.9)	4 (13.3)
Arrondissement 1		68 (16.6)	1 (7.1)	1 (3.5)	0 (0.0)
Arrondissement 4		49 (11.9)	1 (7.1)	1 (3.5)	0 (0.0)
Arrondissement 7		19 (4.6)	0 (0.0)	5 (17.2)	8 (26.7)
Other arrondissements		44 (10.7)	1 (7.1)	9 (31.0)	5 (16.7)
**Main place last 7 days**					
Home		237 (57.7)	8 (57.1)	18 (62.1)	16 (53.3)
Workplace		127 (30.9)	4 (28.6)	5 (17.2)	10 (33.3)
Other places		47 (11.4)	2 (14.3)	6 (20.7)	4 (13.3)
**Place during daytime**					
Saaba		95 (23.1)	5 (35.7)	4 (13.8)	6 (20.0)
Arrondissement 1		75 (18.3)	1 (7.1)	1 (3.5)	0 (0.0)
Arrondissement 10		59 (14.4)	2 (14.3)	2 (6.9)	4 (13.3)
Arrondissement 6		52 (12.7)	2 (14.3)	5 (17.2)	6 (20.0)
Arrondissement 4		49 (11.9)	1 (7.1)	2 (6.9)	0 (0.0)
Arrondissement 7		17 (4.1)	0 (0.0)	5 (17.2)	7 (23.3)
Other places		64 (15.6)	3 (21.4)	10 (34.5)	7 (23.3)

Saaba, Arrondissements 1, 10, 6, 4 and 7 are administrative entities of Ouagadougou, while Saaba is a surrounding rural municipality

^a^Min Minimum age, *Max* Maximum age, *CMA* Medical Centre with Surgical Antenna, *CM* Medical Centre, *CSPS* Primary Healthcare Centre

^b^SD Standard deviation

^c^IQR Interquartile range

While most asymptomatic cases attained tertiary level (6/14), the secondary level of education dominated the other types of dengue. Similarly, most asymptomatic cases were students or private employees (4/14 each), while students predominated among the other types of dengue. Most asymptomatic cases spent their daytime in Saaba (5/14; 35.7%) like participants not infected with dengue (95; 23.1%).

### Knowledge and prevention measures for dengue

The distribution of knowledge and prevention measures is shown in Table 3.

**Table 3:** Distribution of knowledge and prevention measures of dengue according to the type of dengue

Variables	Non-dengue cases (*n* = 411) *n* (%)	Asymptomatic cases (*n* = 14) *n* (%)	Subclinical cases (*n* = 29) *n* (%)	Symptomatic cases (*n* = 30) *n* (%)
**Knowledge category**				
**Knowledge of dengue**				
No	215 (52.3)	4 (28.6)	19 (65.5)	18 (60.0)
Yes	196 (47.7)	10 (71.4)	10 (34.5)	12 (40.0)
**Knowledge of the germ**				
Don’t know	360 (87.6)	7 (50.0)	26 (89.7)	26 (86.7)
Virus	51 (12.4)	7 (50.0)	3 (10.3)	4 (13.3)
**Knowledge of transmission ways**				
Don’t know	118 (28.7)	1 (7.1)	15 (51.7)	16 (53.3)
Mosquito bite	293 (71.3)	13 (92.9)	14 (48.3)	14 (46.7)
**Difference between malaria and dengue**				
No	177 (43.1)	2 (14.3)	16 (55.2)	16 (53.3)
Yes	234 (56.9)	12 (85.7)	13 (44.8)	14 (46.7)
**Dengue prevention measures**				
**Prevention measures**				
No	32 (7.8)	2 (14.3)	2 (6.9)	2 (6.7)
Yes	379 (92.2)	12 (85.7)	27 (93.1)	28 (93.3)
**Vector control measures**				
No	178 (43.3)	6 (42.9)	13 (44.8)	16 (53.3)
Yes	233 (56.7)	8 (57.1)	16 (55.2)	14 (46.7)
**Use of bed nets**				
No	103 (25.1)	4 (28.6)	7 (24.1)	11 (36.7)
Yes	308 (74.9)	10 (71.4)	22 (75.9)	19 (63.3)

The knowledge of dengue reached 71.4% among asymptomatic while most subclinical cases (34.5%) and symptomatic cases (40.0%) did not know about dengue. Similarly, only 10.3% of the subclinical cases and 13.3% of the symptomatic ones knew that dengue is caused by a virus, versus 50.0% of asymptomatic participants. The knowledge of dengue transmission ways was estimated to be 92.9%, 48.3%, and 46.7% among asymptomatic, subclinical, and symptomatic cases, respectively. Participants who knew there was a difference between dengue and malaria represented 44.8% (16/29) and 46.7% (14/30) of subclinical and symptomatic cases, while up to 85.7% of asymptomatic cases knew there was a difference.

Regarding the use of prevention measures, most participants used prevention measures, respectively at 92.2% for non-dengue participants, 85.7% for asymptomatic cases, 93.1% for subclinical cases and 93.3% for symptomatic participants. Except for symptomatic cases (46.7%), most participants (about 55.0%) used vector control measures in the other groups. The use of bed nets reached 70% in the different groups, excluding symptomatic cases (63.3%).

### Symptoms of the subclinical cases

**Fig. 1:**
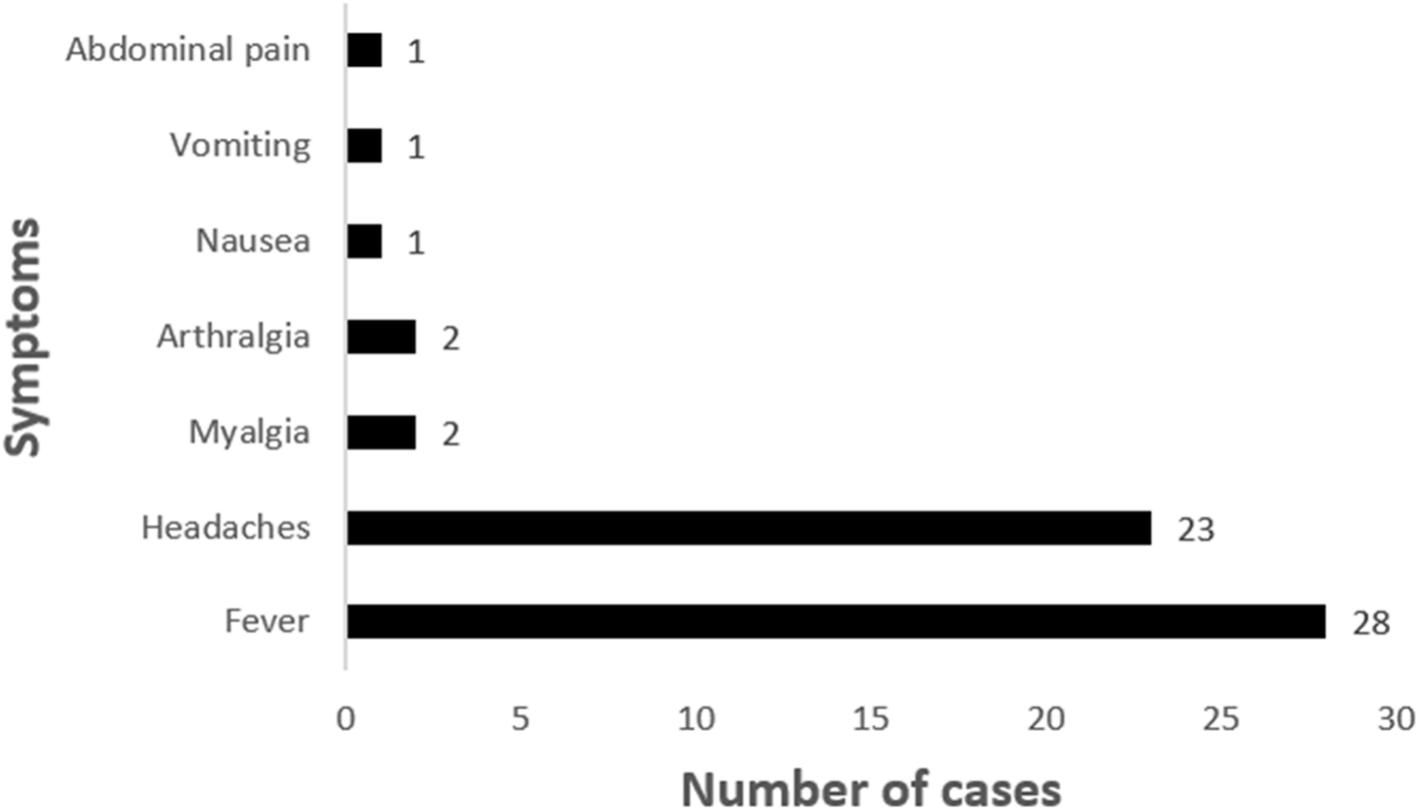
Symptoms experienced by the subclinical cases (*n* = 29)

As per the definition, subclinical cases did not meet the definition of suspected cases by associating fever with 2 of the relevant symptoms. They mostly experienced fever (28/29) and headaches (23/29) (see Fig. 1).

### Factors associated with asymptomatic dengue

Factors associated with asymptomatic dengue are presented in Table 4. From the univariate analysis of the binary logistic regression, the data collection period, the age, education level, knowledge of dengue, its germ, and its transmission ways, being aware that dengue is different from malaria, and cultivated land at less than 50 m from the house, were statistically associated with asymptomatic dengue at the level of 20%. Although not significant at 20%, the sex was input in the multivariable analysis.

**Table 4:** Binary logistic analyses of the factors associated with asymptomatic dengue (Asymptomatic cases vs non-dengue participants; *n* = *425*)

Variables	Univariate binary logistic regression		Multivariable binary logistic regression	
	**Unadjusted *OR* (95% *CI*)**	***P*-value**	**Adjusted *OR* (95% *CI*)**	***P*-value**
**Individual-level factors**				
**Period of collection**				
Non-epidemic year (2022)	1		1	
Epidemic year (2023)	4.29 (0.6–33.2)	0.163	3.9 (0.5–33.0)	0.210
** Age, Years**	1.0 (0.9–1.1)	0.177	1.0 (0.9–1.1)	0.160
**Sex**				
Female	1		1	
Male	1.9 (0.6–5.6)	0.246	1.2 (0.4–3.9)	0.793
**Education level**				
No education	1		1	
Primary	3.7 (0.4–36.6)	0.260	3.1 (0.3–33.4)	0.347
Secondary	1.8 (0.2–16.2)	0.607	1.2 (0.1–14.1)	0.912
Tertiary	6.6 (0.8–56.4)	0.083	4.2 (0.3–53.6)	0.273
**Marital status**				
Never married	1			
Currently married	1.3 (0.4–4.1)	0.691		
Previously married	2.4 (0.5–13.0)	0.305		
**Main occupation**				
Student	1			
Housewife	0.7 (0.1–4.0)	0.698		
Private employee	1.6 (0.4–6.6)	0.524		
Public servant	1.7 (0.3–9.6)	0.558		
Unemployed	1.1 (0.1–10.0)	0.954		
Other occupations	0.3 (0.0–2.7)	0.283		
**Residence**				
Saaba	1			
Arrondissement 6	1.0 (0.2–4.1)	0.948		
Arrondissement 10	0.9 (0.2–3.8)	0.867		
Arrondissement 1	0.3 (0.0–2.6)	0.269		
Arrondissement 4	0.4 (0.1–3.6)	0.419		
Other arrondissements	0.3 (0.0–2.8)	0.300		
**Main place last 7 days**				
Home	1			
Workplace	0.9 (0.3–3.2)	0.911		
Other places	1.3 (0.3–6.1)	0.774		
**Place during daytime**				
Saaba	1			
Arrondissement 1	0.3 (0.0–2.2)	0.215		
Arrondissement 10	0.6 (0.1–3.4)	0.606		
Arrondissement 6	0.7 (0.1–3.9)	0.713		
Arrondissement 4	0.4 (0.0–3.4)	0.393		
Other places	0.7 (0.2–3.0)	0.638		
**Knowledge of the disease**				
No	1		1	
Yes	2.7 (0.9–8.9)	0.093	0.7 (0.1–3.8)	0.646
**Knowledge of the germ**				
Don’t know	1		1	
Virus	7.1 (2.4–21.0)	< 0.001*	5.7 (1.5–22.7)	0.013*
**Knowledge of transmission ways**				
Don’t know	1		1	
Mosquito bite	5.2 (0.7–40.5)	0.113	2.5 (0.2–28.1)	0.454
**Difference between malaria and dengue**				
No	1		1	
Yes	4.5 (1.0–20.5)	0.050	2.1 (0.3–13.2)	0.447
**Household level factors**				
**Animal feces**				
No	1			
Yes	0.5 (0.1–2.2)	0.358		
**Stagnant water**				
No	1			
Yes	0.9 (0.3–3.4)	0.918		
**Hollow containers or used tyres**				
No	1			
Yes	0.9 (0.3–2.6)	0.831		
**Cultivated land less than 50 m away**				
No	1		1	
Yes	2.2 (0.7–6.5)	0.156	2.1 (0.6–7.1)	0.220
**Stretch of water less than 50 m away**				
No	1			
Yes	0.3 (0.0–2.4)	0.267		

*n* = 425; LR chi^2^(11) = 25.3; *P*-value = 0.0082; Pseudo R^2^ = 0.2057; AIC = 121.8; *Significant at 5%

After adjustment and considering the lowest Akaike Information Criterion (AIC) and small *OR* confidence intervals, only the knowledge of the germ transmitting dengue (*P* = 0.013) was significantly associated with asymptomatic dengue fever at 5%. Thus, participants knowing that dengue is transmitted by a virus had a 5.7-fold increase in the chance of having asymptomatic dengue compared to those not knowing.

### Factors associated with subclinical dengue

The factors associated with subclinical dengue are presented in Table 5. From the univariate analysis, subclinical dengue was statistically associated at 5% with data collection period (*P* = 0.030), residence (other arrondissements vs Saaba, *P* = 0.004), place during the daytime (other places vs Saaba, *P* = 0.011), knowledge of dengue transmission ways (*P* = 0.012), presence of stagnant water in the house (*P* = 0.009) and the presence of cultivated land less than 50 m from the house (*P* = 0.027).

**Table 5:** Binary logistic analyses of the factors associated with subclinical dengue (Subclinical cases vs non-dengue participants*; n* = *440*)

Variables	Univariate binary logistic regression		Multivariable binary logistic regression	
	**Unadjusted *OR* (95% *CI*)**	***P*-value**	**Adjusted *OR* (95% *CI*)**	***P*-value**
**Individual-level factors**				
**Period of collection**				
Non-epidemic year (2022)	1		1	
Epidemic year (2023)	9.2 (1.2–68.8)	0.030*	30.2 (2.0– 455.5)	0.014*
** Age, Years**	0.9 (0.9–1.0)	0.598	1.00 (0.9–1.0)	0.888
**Sex**				
Female	1		1	
Male	0.5 (0.2–1.1)	0.074	0.5 (0.2–1.5)	0.208
**Education level**				
No education	1		1	
Primary	0.4 (0.1 – 1.6)	0.198	0.6 (0.1–2.4)	0.429
Secondary	1.9 (0.2 – 16.9)	0.259	0.3 (0.1–1.2)	0.084
Tertiary	6.6 (0.8 – 56.0)	0.402	0.4 (0.1–2.3)	0.327
**Marital status**				
Never married	1			
Currently married	0.7 (0.3–1.5)	0.317		
Previously married	1.2 (0.3–4.4)	0.777		
**Main occupation**				
Student	1		1	
Housewife	1.0 (0.4–2.7)	0.994	0.4 (0.1–1.8)	0.226
Private or public employee	0.5 (0.2–1.6)	0.273	0.3 (0.1–1.2)	0.090
Unemployed	1.7 (0.5–5.9)	0.396	0.8 (0.2–4.1)	0.834
Other occupations	0.4 (0.1–1.3)	0.127	0.3 (0.1–1.3)	0.097
**Residence**				
Saaba	1			
Arrondissement 6	2.8 (0.8–9.9)	0.114		
Arrondissement 10	0.7 (0.1– 4.1)	0.727		
Arrondissement 1	0.4 (0.0–3.4)	0.375		
Arrondissement 4	0.5 (0.1–4.7)	0.552		
Other arrondissements	5.6 (1.8–17.6)	0.004*		
**Main place last 7 days**				
Home	1			
Workplace	0.5 (0.2–1.4)	0.204		
Other places	1.7 (0.6–4.5)	0.297		
**Place during daytime**				
Saaba	1		1	
Arrondissement 1	0.3 (0.0–2.9)	0.308	0.6 (0.1–6.2)	0.644
Arrondissement 10	0.8 (0.1–4.5)	0.806	1.0 (0.1–6.4)	0.964
Arrondissement 6	2.3 (0.6–8.9)	0.233	1.6 (0.4–6.7)	0.530
Arrondissement 4	1.0 (0.2–5.5)	0.972	11.5 (1.0–131.0)	0.049*
Other places (Fada, Loumbila, Koubri)	4.4 (1.4–13.8)	0.011*	4.2 (1.2–14.9)	0.026*
**Knowledge of dengue**				
No	1		1	
Yes	0.6 (0.3–1.3)	0.173	1.7 (0.5–5.4)	0.406
**Knowledge of the germ**				
Don’t know	1			
Virus	0.8 (0.2–2.8)	0.744		
**Knowledge of transmission ways**				
Don’t know	1		1	
Mosquito bite	0.4 (0.2–0.8)	0.012*	0.5 (0.2–1.5)	0.228
**Difference between malaria and dengue**				
No	1			
Yes	0.6 (0.3–1.3)	0.208		
**Household level factors**				
**Animal feces**				
No	1			
Yes	1.6 (0.7–3.5)	0.279		
**Stagnant water**				
No	1		1	
Yes	2. 8 (1.3–6.0)	0.009*	1.8 (0.7–5.1)	0.254
**Hollow containers or used tyres**				
No	1		1	
Yes	1.9 (0.9–4.2)	0.093	1.0 (0.3–3.2)	0.995
**Cultivated land less than 50 m away**				
No	1		1	
Yes	2.4 (1.1–5.1)	0.027*	1.5 (0.6–3.7)	0.362
**Stretch of water less than 50 m away**				
No	1			
Yes	0.5 (0.1–1.6)	0.225		

*n* = 440; LR chi^2^(20) = 43.5; *P*-value = 0.0017; Pseudo R^2^ = 0.2036; AIC = 212.2; *Significant at 5%

After adjustment, the data collection period (*P* = 0.014) and the place during the daytime were associated with increased odds of subclinical dengue. Participants included in 2023 had 30 times higher odds of being subclinical cases than those surveyed in 2022. Participants spending their daytime in Arrondissement 4 (*P* = 0.049) vs those spending the daytime in Saaba had about an 11 times increased chance of being subclinical cases.

### Factors associated with symptomatic dengue

In the absence of any symptomatic case in the non-epidemic year (2022), the variable was not included in the analyses. The binary logistic analyses for symptomatic dengue are found in Table 6.

**Table 6:** Binary logistic analyses of the factors associated with symptomatic dengue (Symptomatic cases vs non-dengue participants*; n* = *441*)

Variables	Univariate binary logistic regression		Multivariable binary logistic regression	
	**Unadjusted* OR* (95% *CI*)**	***P*-value**	**Adjusted *OR* (95% *CI*)**	***P*-value**
**Individual-level factors**				
**Period of collection**				
Non-epidemic year (2022)	N/A		N/A	
Epidemic year (2023)				
** Age, Years**	1.0 (0.9–1.0)	0.169	1.0 (0.9–1.1)	0.877
**Sex**				
Female	1		1	
Male	0.7 (0.3–1.6)	0.390	1.0 (0.4–2.4)	0.927
**Education level**				
No education	1			
Primary	0.6 (0.2–2.6)	0.509		
Secondary	0. 9 (0.3–2.5)	0.826		
Tertiary	1.7 (0.6–4.9)	0.357		
**Marital status**				
Never married	1		1	
Currently married	0.5 (0.2–1.0)	0.060	0.5 (0.1–1.4)	0.160
Previously married	1.0 (0.3–3.6)	0.993	0.6 (0.1–5.1)	0.628
**Main occupation**				
Student	1			
Housewife	1.1 (0.4–3.1)	0.847		
Private or public employee	1.0 (0.4–2.6)	0.932		
Unemployed	1.0 (0.2–4.7)	0.948		
Other occupations	0.5 (0.2–1.8)	0.304		
**Residence**				
Saaba	1			
Arrondissement 6	1.4 (0.4–4.2)	0.595		
Arrondissement 10	0.8 (0.2–3.0)	0.788		
Arrondissement 7	6.0 (2.0–18.6)	0.002*		
Other arrondissements	0.4 (0.1–1.4)	0.175		
**Main place last 7 days**				
Home	1			
Workplace	1.2 (0.5–2.7)	0.713		
Other places	1.3 (0.4–3.9)	0.690		
**Place during daytime**				
Saaba	1		1	
Arrondissement 10	1.1 (0.3–4.0)	0.915	1.7 (0.4–7.7)	0.512
Arrondissement 6	1.8 (0.6–6.0)	0.317	1.8 (0.5–6.5)	0.386
Arrondissement 7	6.5 (2.0–21.8)	0.002*	3.4 (0.9–13.5)	0.085
Other places	0.6 (0.2–1.8)	0.354	0.8 (0.2–2.9)	0.775
**Knowledge of the disease**				
No	1			
Yes	0.7 (0.3–1.6)	0.417		
**Knowledge of the germ**				
Don’t know	1			
Virus	1.1 (0.4–3.2)	0.882		
**Knowledge of transmission ways**				
Don’t know	1		1	
Mosquito bite	0.4 (0.2–0.7)	0.006*	0.6 (0.2–1.5)	0.256
**Difference between malaria and dengue**				
No	1			
Yes	0.7 (0.3–1.4)	0.277		
**Household level factors**				
**Animal feces**				
No	1			
Yes	1.5 (0.7–3.3)	0.335		
**Stagnant water**				
No	1		1	
Yes	2.0 (0.9–4.3)	0.085	1.1 (0.4–3.0)	0.808
**Hollow containers or used t**y**res**				
No	1		1	
Yes	2.4 (1.1–5.2)	0.031*	2.1 (0.7–6.2)	0.175
**Cultivated land less than 50 m away**				
No	1		1	
Yes	2.6 (1.2–5.4)	0.015*	2.8 (1.1–6.9)	0.028*
**Stretch of water less than 50 m away**				
No	1		1	
Yes	0.1 (0.0–1.1)	0.055	0.1 (0.0–0.6)	0.013*

*n* = 441; LR chi^2^(13) = 37, 5; *P*-value = 0.0003; Pseudo R^2^ = 0.1710; AIC = 209.7; *Significant at 5%

From the univariate binary logistic regression, the residence, the place during the daytime, knowledge of transmission ways, hollow containers or used tyres in the house and cultivated land less than 50 m from the house were statistically associated with symptomatic dengue at 5%. After adjustment, participants who reported cultivated land less than 50 m from their house had a three fold increased chance of being symptomatic for dengue (*P* = 0.028). On the contrary, those living less than 50 m from a stretch of water had a reduced chance than those not living near (*P* = 0.013).

## Discussion

This study aimed at estimating the burden of asymptomatic, subclinical, and symptomatic dengue and determining the associated factors among household contacts using a cluster sampling around index cases in 2022 and 2023. As the data collection was household-based and included people who did not seek care for ongoing symptoms or signs, the overall prevalence would represent the total burden of misdiagnosed dengue.

The overall prevalence of dengue reached 15.1% (95% *CI*: 12.0%–18.6%) among the contacts of probable dengue. This prevalence was relatively high and represents the extent of dengue cases escaping the health system. If control measures do not target them, they will constitute reservoirs of dengue virus transmission in the communities. This overall prevalence was higher than what was found in 2013–2014 (8.7%; 33/379) [11] and 2022 (8.2%, 95% *CI*: 6.2%–10.6%) among suspected cases in the Central Region [22]. A lower prevalence was also spotted from perifocal investigations around 149 index cases, with 4.4% dengue, mostly among children (332/346) [28]. From 11 cluster studies, with recruitment in a predefined radius around dengue index cases, the prevalence ranged from 2.2% to 21.5% (median 7.9%) [17]. On the contrary, it was lower than the overall prevalence (25.3%; 740/2929) of probable and confirmed cases between December 2014 and February 2017 in the same region [10]. Dengue among contacts was dominated by the cases presenting some symptoms (12.6%), like subclinical (6.0%) and symptomatic (06.2%) cases, followed by a few asymptomatic cases (2.9%). Poor knowledge of dengue (34.5% and 40%), a virus causing dengue (10.3% and 13.3%), dengue transmission ways (48.3% and 46.7%), and that dengue is different from malaria (44.8% and 46.7%) was usual among subclinical and symptomatic cases. Particularly, symptomatic cases had the lowest use of bed nets (63.3%) and recurse to vector control measures (46.7%). This poor knowledge and the low use of prevention measures could partially explain the high prevalence of subclinical and symptomatic dengue.

Asymptomatic dengue was estimated at 2.9% (95% *CI*: 1.6–4.8%), lower than expected from the literature. A similar study found 7.5% of strictly asymptomatic cases after 2 years of follow-up in Cambodia [28]. A systematic review estimated actual asymptomatic cases at 8% [17]. From the same review, the asymptomatic rate from only cluster studies lay between 7.4% and 92% (median 42%) [17]. These prevalences were consistent with what was pooled from a systematic review in Africa, with 0.0% (95% *CI*: 0.0%–0.5%) for RNA, 3.5% (95% *CI*: 0.8%–7.8%) of IgM and 15.6% (95% *CI*: 9.9%–22.2%) of IgG [18]. However, the asymptomatic prevalence was pooled at 59.3% among family members using missing febrile status as the main criteria [16]. The WHO gives asymptomatic dengue the highest burden of dengue [1]. The discrepancies in asymptomatic dengue burden could be due to differences in the study populations. They could also have to do with the unclear and imprecise definitions of the term asymptomatic, which includes inapparent or clinically undetected infections [17], missing febrile status [16], and underdiagnosed or mild infections [17]. Asymptomatic cases predominantly used prevention measures (85.7%), vector control measures (57.1%) and bed nets (71.4%), which could have contributed to lower the prevalence. They also showed high knowledge of dengue transmission ways (92.9%) and the fact that dengue differs from malaria (85.71%). Beyond that, it is necessary to know the actual extent of asymptomatic dengue, as such cases can transmit dengue to mosquitoes despite their lower levels of viremia [29]. By the way, asymptomatic cases are more infectious to mosquitoes than symptomatic ones at a given level of viremia [29].

From this sample, IgM alone or associated with other markers was the most prevalent marker overall (60.2% in total) and among all types of dengue. IgM is a marker of a recent primary infection; coupled with IgG, it is still a recent primary infection or a late secondary infection. On the contrary, a study that included febrile participants found a high proportion of AgNS1 (11%) and a low proportion of IgM (4%), though [10]. Delayed contact with the participants would explain the predominance of IgM markers. Finally, these participants did not seek care and passed the acute stage (AgNS1), when they could be infectious to mosquitoes. Moreover, this health-seeking behavior is risky as complications could quickly arise.

Studies revealed that dengue does not vary with the types of dengue [29]. In this study, only serotype 3 was confirmed among symptomatic (7/10 confirmed cases) and subclinical (3/10 confirmed cases) cases. Then, it was not possible to determine whether dengue virus serotype 3 was associated with a specific type of dengue. Also, given the small number of confirmed cases by RT-PCR, we cannot conclude if serotype 3 predominated during the 2023 epidemic. However, dengue incidence varies biannually in the country [30], probably due to a shift in dominant serotypes over time [31]. Thus, although all 4 serotypes circulate in the country, the dominant serotypes appear to vary over time [11, 12]. In 2013–2014, if serotypes 2, 3 and 4 were identified to circulate in the Central Region, serotypes 2 and 3 were more frequent, and it seemed serotype 3 predominated [10, 11, 32]. For the 2016–2017 epidemic, despite the co-circulation of serotypes 2 and 3, serotype 2 was identified as dominant [10, 33]. The primary infection of a serotype procures complete immunity for it but not for the other serotypes [34]. With the shift in predominant serotypes, populations may be subject to the phenomenon of antibody-dependent enhancement (ADE) with an immunologic response to the second serotype without neutralizing the virus. Such a phenomenon increases the risk of severe dengue and death.

The factors associated with the different types of dengue were also assessed and were found to differ according to the type of dengue. For the asymptomatic cases, their likelihood of being infected was associated with knowing a virus transmits dengue, which was surprising. Enrolling in the study in 2023 and spending the daytime at Arrondissement 4 increased the odds of being a subclinical case. Similarly, dengue suspected cases residing or staying daytime at Arrondissement 4 were more likely to test positive for dengue RDT in 2022 in the Central Region, Burkina Faso [22, 35]. In effect, Arrondissement 4 is a wet and wooded environment with two dams and the Bangr-Weogo Park, ensuring favorable conditions for mosquito breeding and development. It explains why participants living less than 50 m from cultivated land had increased odds of being symptomatic cases. However, participants living near a stretch of water had reduced odds of being symptomatic cases. In fact, the immature stages of *Aedes* mosquitoes mostly develop in artificial habitats such as water tanks (45.2%), waste left in the house (24.7%) and tyres (21.6%) [11]. Used tyres were indeed the most common larval breeding sites in urban areas, while in peri-urban and rural areas, drinking troughs and water storage containers were the most frequent sites [36, 37].

This study gives insights into the burden of the different types of dengue from a population-based perspective. However, some limitations can go with the way participants were selected. If that allows epidemiological linkages with the index cases, it will likely overestimate the dengue burden. So, the prevalence of the different types of dengue in the true population could be lower than what was estimated in this study. Despite the cluster sampling, the prevalence of asymptomatic dengue was lower than expected by the WHO. Using RDTs instead of ELISA could also be limiting, as ELISA carries more sensitivity and specificity. This study could be the first to estimate the burden of the different types of dengue in Burkina Faso and will help understand the epidemiology of the disease.

## Conclusions

It is crucial for the surveillance system to capture the actual epidemiology of dengue. This study found that several cases are missed by the health system in Burkina Faso, including symptomatic ones. Poor knowledge and low use of preventive measures were found among symptomatic and subclinical cases, explaining probably the high burden. Asymptomatic dengue was rather low, partially due to the fair knowledge of dengue and the high use of preventive measures. In addition, serotype 3 could have originated the 2023 epidemic. To control the disease, interventions should target contacts of dengue cases as well as raise awareness of the disease and the use of preventive measures. Population studies are welcome to provide more accurate estimates of the dengue burden in the country.

## Data Availability

The datasets generated and/or analyzed during the current study are available from the corresponding author on reasonable request.

## Abbreviations

AgNS1: Antigen nonstructural protein 1
CM: Medical centre
CMA: Medical Centre with Surgical Antenna
CSPS: Primary healthcare centre
DENV: Dengue virus
IgG: Immunoglobulins G
IgM: Immunoglobulins M
RDT: Rapid diagnostic test
WHO: World Health Organization
